# Development of a Care Labelling Process for Compression Stockings Based on Natural (Cotton) Fibers †

**DOI:** 10.3390/polym13132107

**Published:** 2021-06-26

**Authors:** Cevza Candan, Banu Nergis, Sena Cimilli Duru, Bilge Koyuncu

**Affiliations:** Faculty of Textile Technologies and Design, Istanbul Technical University (ITU), Istanbul 34467, Turkey; candance@itu.edu.tr (C.C.); uygunf@itu.edu.tr (B.N.); cimilli@itu.edu.tr (S.C.D.)

**Keywords:** medical compression stocking, cotton, nylon, care labelling

## Abstract

This study is to investigate to what extent the performance of compression stockings with cotton components deteriorates after repeated washing processes. Four compression stockings having at least one cotton constituent yarn and two all-nylon stockings as reference samples were produced under controlled commercial conditions. After analysing the data obtained, a care labelling process for the compression socks with cotton components was developed such that they can preserve their compression properties over successive laundering treatments.

## 1. Introduction

It has been estimated that 2% of the general population in the world is suffering from chronic venous disorders. This rate is further increased up to 4% in people over the age of 65. This implies a significant socioeconomic impact on the total healthcare budgets of developed countries in order to treat millions of patients every year [1]. This number is expected to rise with an aging population and the changing lifestyles of people all over the world, which at the same time suggests that there is a sizable market in compression products. [2,3].

As an efficient and long-term medical intervention for chronic venous disorders, compression therapy is preferred so as to minimize post-thrombotic syndrome, prevent ulcer recurrence, and alleviate related symptoms, such as leg pain and swelling caused by damaged veins [4,5,6]. With compression therapy, the problematic body part is subjected to external pressure from medical textile-based products such as compression stockings for legs [7,8]. The effectiveness of the compression treatment is very much dependent on the interface pressure between the textiles and the skin, and the literature shows that factors such as limb shape, size and properties of compression products do have a significant influence on this pressure [9,10,11]. The changes occurring in the interface pressure applied by a medical compression product is, on the other hand, quite important because such changes directly affect the potency of the treatment of the compression garment [5,12,13,14,15]. In that respect, it should be noted that the success of compression treatment is very much determined by the fiber and fabric construction employed in a product. Medical compression stockings are used to provide maximum compression at the ankle. This compression gradually decreases as the limb circumference increases. Therefore, prescribing the appropriate size and compression rating for the patient being treated is essential to having the maximum benefit from the product. A general guide to the amount of compression recommended for various venous disorders is given in Table 1 [16].

Yarns used for medical compression garments, including stockings, can be produced by various covering methods such as single or double covering, core spinning, and air jet covering [11]. Core–sheath yarns which are composed of the elastomeric filament covered by staple cotton, PET, or nylon-based fibers are commonly preferred in the medical compression products market. However, it is also possible to employ the elastomeric yarn in the form of weft inlay in the knitted fabric structure with the help of different stitch types, such as tuck and miss-stitch (float). The structure, together with material properties (i.e., fiber fineness, covering yarn count, and type of covering, etc.), can be optimized to satisfy the required criteria in terms of degree of compression, circumferential dimensions, weight, thickness, extensibility, etc., from a compression garment. Medical compression stockings should be designed such that they can maintain a uniform interface pressure gradient over the limb for faster and more effective recovery results. However, because of the viscoelastic nature of textiles, the stress developed in a medical stocking under constant extension reduces over time. This process is called “stress relaxation” on which successive washing may also have an effect [17,18,19,20,21]. Synthetic fibers (e.g., nylon, PET, elastane) have low stress relaxation in comparison to cotton fiber because of their higher relaxation time, whereas elastomeric yarns keep the internal stress for a longer time because of their good elastic properties. This is one of the reasons why elastomeric yarns are utilized for medical stockings and other compression garments [12,13,22,23].

As compression stockings have to be worn almost all day long, they need to be washed almost every day for hygiene issues. In that respect, they are expected to retain not only their shape, but also their compression level. The literature survey reveals that studies on the effects of factors such as washing and multiple wearing on the life span of compression stockings are mainly confined to commercial products and those from synthetic fibers [17,18,19,20,21]. Furthermore, for the medical stockings from synthetic-based yarns, irritation and rashes are among the most common skin problems, and the use of natural fibers in such products may help to overcome such skin problems. Accordingly, this study was conducted to develop a care labelling process for the compression stockings based on natural fibers, namely, cotton. Knee-high compression stockings having cotton covered yarn that could meet the requirements of Class III compression for patients having sensitive skin were developed and produced. Dimensional and compression performance changes in the samples that were repeatedly hand- and machine-washed were studied. Finally, the care labelling directions for such stockings were suggested.

## 2. Materials and Methods

**Figure 1:**
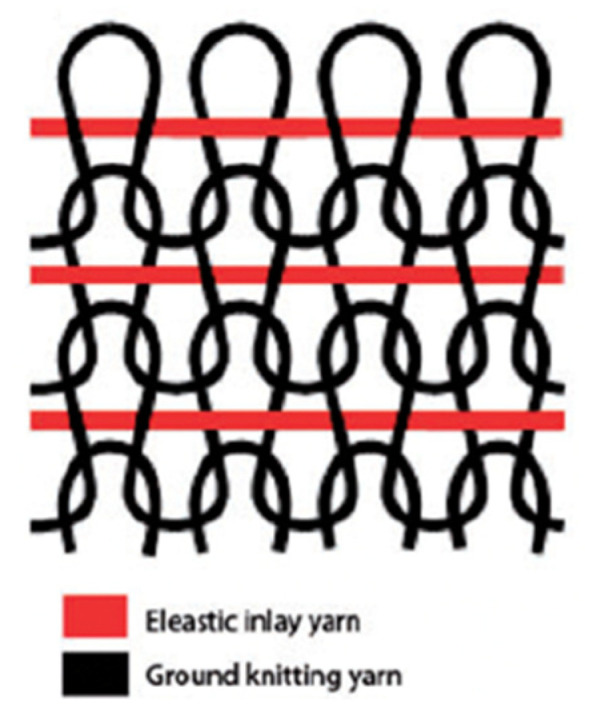
Fabric structure [11].

A total of six types of stockings for a fixed leg size exhibiting compression levels of Class III (34–46 mmHg) were designed and produced in the form of a plain jersey structure in which the elastic yarns are integrated in as weft inlay under controlled commercial conditions (Figure 1). For knitting, Merz (Merz Maschinenfabrik GmbH, Hechingen, Germany) Model: CC4–II8 single-cylinder circular knitting machine—which is 4 ¾ inches in diameter and equipped with 420 needles—was used. The inlay and ground yarns were inserted in the fabrics at the constant input tensions of 1.8 cN and 2.6 cN, respectively. Variteks Ortopedi Sanayi A.Ş, Istanbul, Turkey offers seven sizes for the knee-high compression stockings depending on the tendon circumference: size 1 (30–32), size 2 (32–34), size 3 (34–36), size 4 (36–38), size 5 (38–40), size 6 (40–42), and size 7 (42–45).

For the work, size 4 was selected because it is more representative in terms of the number of patients using such stockings globally. The details of the double-covered yarns employed in the production are given in Table 2.

**Figure 2:**
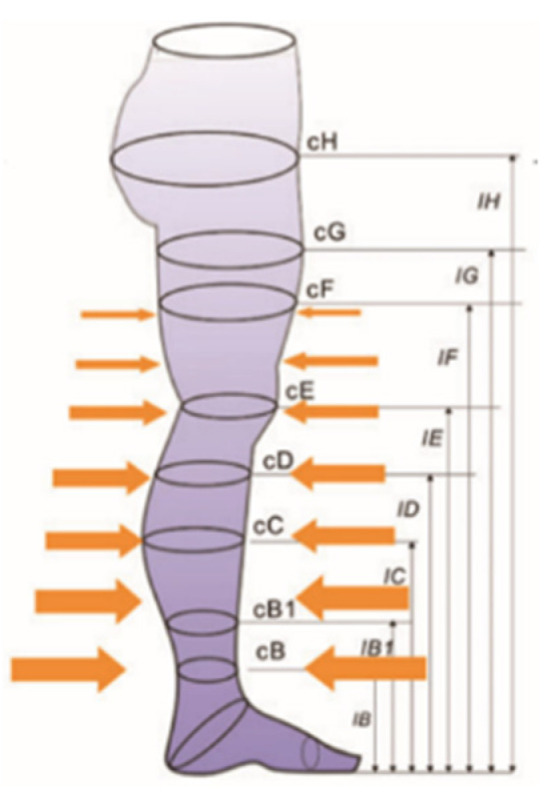
Measurement Points of the Stockings [24].

During the production of the samples, a colored yarn is fed to the machine to mark the measurement points at B, B1, C and D (Figure 2). Three pairs from each combination of yarns were knitted and grouped as:

*Untreated:* No washing and ironing was applied (greige state) to the stockings.

*Ironed*: The stockings were ironed using Alba Hosiery Industrial Iron (Alba Ütüleme Sistemleri, İstanbul, Turkey).

*Hand-washed:* The stockings were hand-washed at 25 °C in tap water using commercial soap powder for 5 min. The water was drained by placing the samples between towel layers for 3–5 min, and then the samples were flat dried.

*Machine-washed:* The stockings were machine-washed using Bosch Avantixx 7 Varioperfect (BSH Hausgeräte GmbH, Munich, Germany) with Sensitive/Silk/Delicate Textiles Laundering Program (30 °C cold washing, spin speed 600 rev/min) and they were then flat dried.

**Figure 3:**
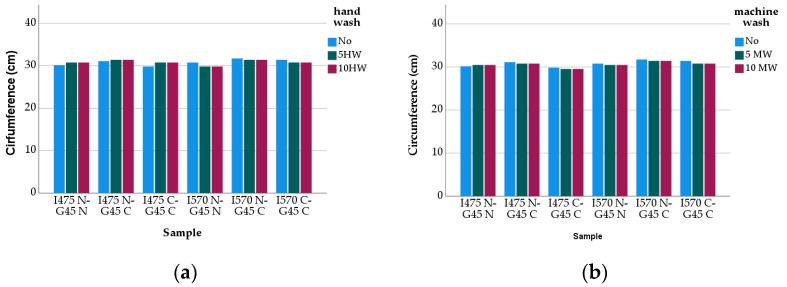
Circumference Change: (**a**) Hand-wash vs. (**b**) Machine-wash.

**Figure 4:**
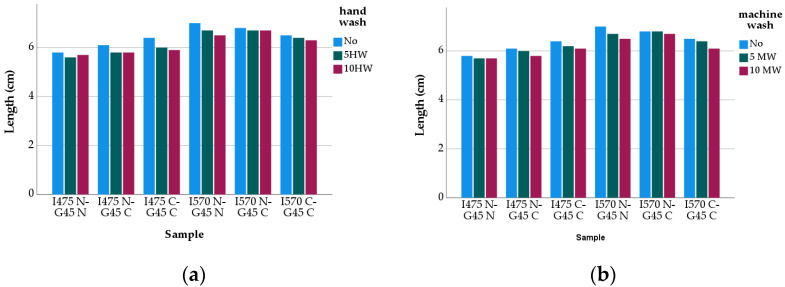
Length Change: (**a**) Hand-wash vs. (**b**) Machine-wash.

After the hand- and machine-washing processes, the samples were relaxed and conditioned for 24 h under the standard atmospheric condition (relative humidity 65 ± 5%, 21 ± 2 °C). Then, they were worn for 20 min before starting the next washing cycle. For each group of stockings, the relevant measurements were taken from the samples and the results are given in Figure 3 and Figure 4.

**Figure 5:**
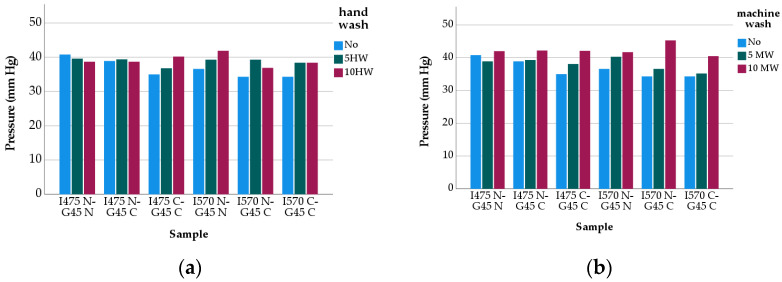
Compression Pressure Change: (**a**) Hand-wash vs. (**b**) Machine-wash.

The samples were tested for their compression performance in accordance with RAL-GZ 387/1-2008 Quality and Test Specifications for medical compression hosiery, which is prepared by RAL German Institute for Quality Assurance and Labelling [25]. The MST Professional 2 medical stocking tester (SwissLastic AG St. Gallen, Gallen, Switzerland) was employed for this purpose. The tester comprises a variable leg form that covers 95% of known sizes and a flat measuring probe that enables easy mounting of the stocking to be tested onto the leg form without overstretching. A compression profile is obtained by the measurement of all measuring points [26]. Since “millimeters of mercury” (mm/Hg) is a more widespread unit of measurement in the textile industry than the “kilo pascal” (kPa) and it exactly indicates the pressure, regardless of the yarn and the “weight” of the yarn, “millimeters of mercury” (mm/Hg) was employed in the study. The data obtained is given in Figure 5.

The statistical evaluation of the data was performed with the SPSS 27 software package (IBM SPSS Statistics, New York, USA). One-way analysis of variance (ANOVA) was employed and the factors were considered to be significant at a *p*-value of less than 0.05. Additionally, *t*-test was used to evaluate the results of the unwashed, 5 times washed and 10 times washed compression stockings. In addition, multiple regression analysis was conducted for describing the relationship between the changes in the fabric properties and the investigated parameters (Table 3, Table 4 and Table 5).

## 3. Results and Discussion

### 3.1. Multiple Washing Effect on Circumference Changes

Figure 3 represents the circumference changes in the compression stockings before and after washes. The circumference values of the samples were slightly affected by the yarn and washing types, and the pattern of the change was not regular, though it appeared that the circumference values of the washed stockings were smaller than those of the unwashed stockings (i.e., shrinkage occurred).

The effect of the variables (type of cover yarns, inlay elastane yarn count) on the circumference of the unwashed stockings was evaluated by multiple linear regression, and it was found that all selected variables were highly significant factors affecting the resultant circumference with an R-square value of 0.877 (sig. 0.000). The expression of multiple linear regression model as follows (Table 3):

Circumference = 28.903 + 0.938X − 0.798Y + 0.953Z(1)

demonstrated that the most influential factor on the circumference is the second cover yarn type of ground yarn, and it is followed by the yarn count of the inlay elastane and the second cover yarn type of inlay elastane. Furthermore, using one-way ANOVA, it was demonstrated that the yarn-related variables (fiber and yarn type, yarn count) were statistically significant in governing the circumference values of the stockings (F (5,12) = 159,291; sig. 0.000; *p* < 0.05). For the unwashed stockings, although the samples having 570 dtex inlay elastane yarn gave slightly higher circumference values (Figure 3), the difference was found to be statistically significant (t = −3.915, sig. 0.001). Although it seemed to be to a lesser extent than that for the length of the stockings, elongation behavior of the inlay yarns (Table 1) seemed to be reflected on the circumference of the socks. When the second cover yarn of the inlay yarns is concerned, the samples made from 33–24 f nylon and 80/1 Ne cotton did not depict a marked difference (t = 0.929, sig. 0.367). A similar result was also obtained for the effect of the second cover yarn of the ground yarn from 40–44 f nylon and 80/1 Ne cotton on the circumference of the samples (t = −1.693, sig. 0.110). This may suggest that the yarn type, together with the fiber type and the elongation performance of the yarns, did have a holistic reflection on the circumference values of the stockings. It is also worth noting that depending on highly elastic properties of the stockings, deformations occur more than one direction.

The circumference values of the washed stockings revealed that, irrespective of the washing type, there was no statistically significant change between the washed and unwashed values up to five cycles. However, when the number of washes were increased to 10, the circumferences of the stockings did decrease and, according to the paired *t*-test conducted, the difference between the washed and unwashed samples became significant (t = 13.086 sig. 0.00 for 10 times hand-washed; t = 12.039 sig. 0.000 for 10 times machine-washed). The statistical analysis also indicated that increasing the number of washes from 5 to 10 resulted in a significant difference between the circumference values for both hand and machine-washes (t = 17.659 sig. 0.000).

### 3.2. Multiple Washing Effect on Length Changes

Figure 4 shows that the B-B1 length values of the samples were affected by the inlay elastane yarn count such that the stockings made from 475 dtex inlay elastane yarn consistently gave lower values (with a statistically significant difference, t= −5.547 sig. 0.000) when compared to the other group of samples from 570 dtex. The inlay yarns were included in the structures at the same pre-tension. However, as may be seen from Table 2, the elongation performance of these yarns is different which may have affected the length values of the stockings in such a way that the strain energy on the 475 dtex inlay yarns stored by pre-tensioning released less effectively after knitting, causing the samples from them to retract and settle in lower lengths. It also needs to be noted that the stockings from 570 dtex inlay elastane yarn tended to be shorter as the cotton content increased, whereas an opposite tendency was observed for the stockings from 475 dtex inlay elastane yarn. This may be due to the combined effect of cover yarn type, fiber type and yarn elongation on the lengthwise dimensions of the stockings (Table 2), as it appears that cotton is not the only variable influencing this very behavior. Moreover, as was expected, the length values of the washed stockings consistently decreased as the number of washing cycles was increased, though the drop in the length values for the first five washes was slightly higher.

The multiple linear regression analysis of the effect of the variables (fiber type for cover yarns, inlay elastane yarn count) on the B-B1 length values of the unwashed stockings gave the following function (R-square = 0.661, sig. 0.001) (Table 4):

B-B1 Length = 5.350 + 0.667X − 2.823E-17Y + 0.050Z(2)

As may be seen from the function, the most influencial factor on the length is Yarn Count of Inlay Elastane. One-way ANOVA analysis showed that the yarn related variables (fiber & yarn type, yarn count) were influencial on the length changes of the stockings (F(5,12) = 58,400 sig. 0.000; *p* < 0.05).

The analysis of the length values of the washed stockings revealed that there was a significant difference between the values of the washed and unwashed samples (t = 8.702 sig. 0.00 for 5 times hand-washed; t = 5.831 sig. 0.00 for 5 times machine-washed; t = 6.974 sig. 0.00 for 10 times hand-washed; t = 7.988 sig. 0.000 for 10 times machine-washed), and the length of the samples tended to decrease as the number of washes increased. The statistical analysis also showed that the difference between the length values of the 5 and 10 times washed samples were significant, irrespective of washing type (t = 2.153 sig. 0.046 for hand-washed, t = 6.460 sig. 0.000 for machine-wash).

### 3.3. Multiple Washing Effect on Compression Pressure Changes

Figure 5 represents the compression pressure values at the ankle of the samples before and after washes. As may be seen from these, the samples satisfied the requirements of Class III, irrespective of material content, yarn count, and washing type. Figure 5 also shows that up to five washing cycles, the influence of washing type on the compression pressure is not as high as expected, though the intensity of the compression pressure is more significant at the end of ten cycles of machine-washing, in comparision to ten cycles of hand-washing.

According to the multiple linear regression analysis, the effect of the factors under discussion on the compression pressure of the unwashed stockings was studied and the analysis revealed the following function (R-square = 0.871, sig. 0.000), which demonstrates that the most influential factor on the compression pressure is yarn count of the inlay elastane, Second Cover Yarn Count of Ground Yarn and Second Cover Yarn Count of Inlay Elastane, in turn (Table 5).

Compression Pressure = 47.500 − 3.167X − 1.950Y − 2.100Z(3)

The one-way ANOVA test also implied that there was a significant difference between all the variables studied (F(5,12) = 2163,300; *p* < 0.05).

For unwashed stockings, namely ironed ones, it was found that there was a statistically significant difference between the compression pressures of the stockings from inlay elastane yarns of 475 dtex and 570 dtex (t = 3.381 sig. 0.004), and the samples having 475 dtex inlay elastane yarn gave higher compression pressures (Figure 5). Although socks having coarser inlay elastane are expected to yield higher pressure values, the result obtained can be attributed to the differences in the circumference and length (B-B1) values of the samples. The samples from 475 dtex inlay elastane yarn did have much lower circumference and length values than the samples from 570 dtex inlay elastane yarn, which affected the ability of the material to stretch, and in turn, the pressure exerted. In addition to that, the compression level may have also been influenced by the other covering variables, namely fiber type and yarn count of covering yarns, though the yarn count of inlay elastane is the most critical factor governing compression pressure in medical socks. So far as the second cover yarn of the inlay yarns is concerned, the samples made from 33–24 f nylon (Figure 5) gave higher compression pressure values than the others having 80/1 Ne cotton (t = 2.893 sig. 0.012). Finally, the compression pressure of the samples from the second cover yarn of the ground yarn from 40–44 f nylon was also found to be greater than that from 80/1 Ne cotton. Moreover, the difference was statistically significant (t = 2.931 sig. 0.01). With reference to these findings, it may be concluded that the presence of cotton in the yarns, as the second cover and/or both the first and second covers, caused a decrease in the compression pressure values, which are still in the acceptable range for Class III requirements. This may be due to the fact that the cotton content in the yarns enhances the yarn-yarn friction and thus diminishes the possibility of the samples to dry relax and have increased geometrical characteristics for better compression pressures.

The compression pressure values of the washed stockings revealed that the intensity of the compression pressure increased and, furthermore, there was a significant difference between the compression pressure values of the washed and unwashed samples (t = 4.234, sig. 0.01 for 5 times hand-washed; t = −3.113, sig. 0.006 5 times machine-washed; t = −3.692, sig. 0.002 for 10 times hand-washed; t = −7.581, sig. 0.000 to times for machine-washed). The statistical analysis also showed that unlike the hand-washed samples (t = −0.677 sig. 0.507), the difference between the compression pressure values of the 5 and 10 times machine-washed samples were significant (t = −7.531 sig. 0.000). Finally, the effect of washing type on the compression pressure of the stockings were analysed, and no statistically significant difference between these values was observed for the first five washes (t = 1.771, sig. 0.095). On the other hand, when the number of the wash cycles is increased up to ten (10), the tendency did change (t = −4.959, sig. 0.000). These results, together with the changes in the samples’ circumferences and lengths, may suggest that washing relaxation of the stockings did have a positive impact on the compression pressure values. As the number of washes increased, the compression pressures of the samples increased (Figure 3). As a final word, it may be stated that machine-washing (30 °C cold washing, 600 rev/min spin speed, no tumble drying), can be recommended for caring of medical compression stockings.

## 4. Conclusions

The present study investigated the role of different fiber materials (i.e., nylon and cotton) and inlay elastane yarn count in the compression pressure performance of stockings designed for a fixed leg size, which exhibited compression levels of Class III (34–46 mmHg) and were produced in the form of a plain jersey structure in which the inlay elastane yarns were integrated in as weft inlay under controlled commercial conditions. In doing so, more emphasis was given to the change of the pressure performance of the stockings depending on the washing regimes, namely, hand-washing and machine-washing. Based on the results, it may be concluded that:

- The samples satisfied the requirements of Class III, irrespective of material content (cotton–cotton, cotton–nylon, or nylon–nylon), which means that without compromising the compression levels, cotton yarns could have been employed together with nylon yarns in the compression stockings for enhancing their comfort properties.
- The compression pressure values of the washed stockings demonstrated that the intensity of the compression pressure increased and, furthermore, there was a significant difference between the compression pressure values of the washed and unwashed samples. Moreover, the analysis of the effect of washing type on the compression pressure of the stockings presented no statistically significant difference between the value sets of hand and machine-washed samples for the first five washes, whereas the tendency did change when the number of the wash cycles was increased up to ten. Therefore, it may be concluded that the washing processes (hand- or machine-wash) caused dimensional shrinkage in the compression stockings, which helped them to maintain their original degree of compression.

Finally, medical compression stocking producers generally recommend hand-washing for the care of such products. However, the study suggested delicate machine-washing can be offered as a better alternative as it provides better compression levels, though further research is needed for investigating the effect of more than ten cycles of machine-washing on the life span of stockings in terms of viscoelastic behaviors of the materials involved.

## Figures and Tables

**Table 1:** Recommended degree of compresion [16].

Degree of Compression (kPa)	Venous Disorders
<2.6	Prevention of deep vein thrombosis, mild edema, aching legs
2.6–4	Mild varicose veins, mild to moderate edema, long-haul flights, varicose veins related to pregnancy
4–5.3	Venous ulcers, deep vein thrombosis, superficial thrombophlebitis, viscose veins with severe edema, post-thrombotic syndrome, mild lymphedema
>5.3	Severe lymphedema, severe chronic venous insufficiency

**Table 2:** Details and Elongation of Double covered yarns under fixed load.

Yarn Code1	Yarn Count (Core/1st Cover/2nd Cover)	Elongation (%) under 100 g
I475-N	475 dtex Elastane/33/24 f Nylon/33/24 f Nylon	331
I475-C	475 dtex Elastane/33/24 f Nylon/80/1 Cotton	403
I570-N	570 dtex Elastane/78/24 f Nylon/78/24 f Nylon	370
I570-C	570 dtex Elastane/78/24 f Nylon/80/1 Cotton	389
G45-N	45 dtex Elastane/40/44 f Nylon/40/44 f Nylon	219
G45-C	45 dtex Elastane/40/44 f Nylon/80/1 Cotton	270

1 I: Inlay Yarn, G: Ground Yarn.

**Table 3:** Estimated effects of the variables on circumference changes.

Term	Notation	Coefficient	Std. Error of Coefficient	*p*
Constant	-	28.903	0.307	0.000
Yarn Type of Inlay Elastane	X	0.938	0.125	0.000
Second Cover Yarn Type of Inlay Elastane	Y	−0.798	0.154	0.000
Second Cover Yarn Type of Ground Yarn	Z	0.953	0.154	0.000

**Table 4:** Estimated effects of the variables on length changes.

Term	Notation	Coefficient	Std. Error of Coefficient	*p*
Constant	-	5.306	0.313	0.000
Yarn Type of Inlay Elastane	X	0.667	0.128	0.000
Second Cover Yarn Type of Inlay Elastane	Y	−2.823 × 10^−17^	0.157	1.000
Second Cover Yarn Type of Ground Yarn	Z	0.050	0.157	0.754

**Table 5:** Estimated effects of the variables on compression changes.

Term	Notation	Coefficient	Std. Error of Coefficient	*p*
Constant	-	47.5000	1.152	0.000
Yarn Type of Inlay Elastane	X	−3.167	0.470	0.000
Second Cover Yarn Type of Inlay Elastane	Y	−1.950	0.576	0.004
Second Cover Yarn Type of Ground Yarn	Z	−2.100	0.576	0.003

## Data Availability

The data presented in this study are available on request from the corresponding author.

## References

[1] Pascarella L., Shortell C.K. (2015). Medical management of venous ulcers. Semin. Vasc. Surg..

[2] Gupta D. (2011). Functional Clothing-Definition and Classification. Indian J. Fibre Text. Res..

[3] Bergan J.J., Schmid-Schönbein G.W., Smith P.D.C., Nicolaides A.N., Boisseau M.R., Eklof B. (2006). Chronic venous disease. N. Engl. J. Med..

[4] Partsch H. (2013). Compression therapy in leg ulcers. Rev. Vasc. Med..

[5] Partsch H. (2014). Compression for the management of venous leg ulcers: Which material do we have?. Phlebology.

[6] Mosti G., Picerni P., Partsch H. (2012). Compression stockings with moderate pressure are able to reduce chronic leg oedema. Phlebology.

[7] Cullum N.A., Nelson E.A., Fletcher A., Sheldon T. (2000). Compression for Venous Leg Ulcers. Cochrane Database Syst. Rev..

[8] Kumar B., Das A., Alagirusamy R. (2014). Science of Compression Bandages.

[9] Partsch H., Clark M., Bassez S., Benigni J.-P., Becker F., Blazek V., Caprini J., Cornu-Thénard A., Hafner J., Flour M. (2006). Measurement of lower leg compression in vivo: Recommendations for the performance of measurements of interface pressure and stiffness. Dermatol. Surg..

[10] Hirai M. (1998). Changes in interface pressure under elastic and short-stretch bandages during posture changes and exercise. Phlebology.

[11] Liu R., Guo X., Lao T.T., Little T. (2017). A critical review on compression textiles for compression therapy: Textile-based compression interventions for chronic venous insufficiency. Text. Res. J..

[12] Kumar B., Das A., Alagirusamy R. (2012). Prediction of internal pressure profile of compression bandages using stress relaxation parameters. Biorheology.

[13] Kumar B., Das A., Alagirusamy R. (2013). An approach to examine dynamic behavior of medical compression bandage. J. Text. Inst..

[14] Kumar B., Das A., Alagirusamy R. (2013). Effect of material and structure of compression bandage on interface pressure variation over time. Phlebology.

[15] Mosti G., Partsch H. (2010). Inelastic bandages maintain their hemodynamic effectiveness over time despite significant pressure loss. J. Vasc. Surg..

[16] Hu J., Kumar B., Lu J. (2020). Chapter 27: Fibers for Medical Compression. Handbook of Fibrous Materials.

[17] Siddique H.F., Mazari A.A., Havelka A., Kus Z. (2019). Performance Characterization of Compression Socks at Ankle Portion under Multiple Mechanical Impacts. Fibers Polym..

[18] Maleki H., Aghajani M., Sadeghi A.H., Jeddi A.A. (2011). On the Pressure Behavior of Tubular Weft Knitted Fabrics Constructed from Textured Polyester Yarns. J. Eng. Fibers Fabr..

[19] Siddique H., Mazari A., Havelka A., Kus Z. (2020). Performance Characterization and Pressure Prediction of Compression Socks. Fibers Polym..

[20] Gohar E.S., Mazari A. (2020). Effect of Multiple Use on the Durability of Compression Socks. Fibres Text..

[21] Harpa R., Cristina P., Cezar-Doru R. (2010). A New Approach for Testing Medical Stockings. Text. Res. J..

[22] Das A., Kumar B., Mittal T., Singh I., Prajapati S. (2012). Pressure profiling of compression bandages by a computerized instrument. Indian J. Fibre Text. Res..

[23] Senthilkumar M., Anbumani N., Hayavadana J. (2011). Elastane Fabrics—A Tool for Stretch Applications in Sports. Ind. J. Fibre Tex. Res..

[24] Wang Y., Zhang P., Zhang Y. (2014). Experimental investigation the dynamic pressure attenuation of elastic fabric for compression garment. Text. Res. J..

[25] Medical Compression Hosiery. Hosiery Quality Assurance: RAL-GZ 387/1. https://www.gzg-kompressionsstruempfe.de/uploads/media/RAL_GZ_387_englische_Version.pdf.

[26] MST Professional 2 Medical Stocking Tester Catalogue. https://www.swisslastic.ch/files/Druckmessgeraete/MST-professional%202-EN_ES_JP_CN-2013-4p.pdf.

